# Right Ventricular Structure and Function in Patients with Primary Aldosteronism: A Cardiac Magnetic Resonance Study

**DOI:** 10.3390/jcm14155367

**Published:** 2025-07-29

**Authors:** Mateusz Śpiewak, Sylwia Kołodziejczyk-Kruk, Agata Kubik, Agnieszka Łebek-Szatańska, Elżbieta Szwench-Pietrasz, Elżbieta Florczak, Magdalena Januszewicz, Andrzej Januszewicz, Magdalena Marczak

**Affiliations:** 1 Magnetic Resonance Unit, National Institute of Cardiology, 04-628 Warsaw, Poland; akubik@ikard.pl (A.K.); mmarczak@ikard.pl (M.M.); 2 Department of Hypertension, National Institute of Cardiology, 04-628 Warsaw, Poland; skolodziejczyk@ikard.pl (S.K.-K.); alebek@ikard.pl (A.Ł.-S.); eszwench@ikard.pl (E.S.-P.); eflorczak@ikard.pl (E.F.); ajanuszewicz@ikard.pl (A.J.); 3 Second Department of Clinical Radiology, Medical University of Warsaw, 02-091 Warszawa, Poland; magdalena.januszewicz@wum.edu.pl

**Keywords:** cardiac magnetic resonance, hypertension, primary aldosteronism, right ventricle, strain

## Abstract

**Background/Objectives:** While aldosterone excess has a detrimental impact on the left ventricle, no data exist concerning right ventricular (RV) function in primary aldosteronism (PA) patients. We aimed to assess RV structure and function in patients with PA using cardiac magnetic resonance imaging. **Methods:** Thirty PA patients and 30 age- and sex-matched healthy volunteers were studied. All patients underwent cardiac magnetic resonance with the assessment of RV structure and function. **Results:** Neither the RV mass index (RVMi) nor the RV ejection fraction (RVEF) correlated with the aldosterone levels (*p* = 0.36 and *p* = 0.37, respectively). On the contrary, we found a weak positive correlation between the RV end-diastolic volume index (RVEDVi) and aldosterone concentration (rho = 0.5, *p* = 0.005). Neither the RVEDVi nor the RVEF differed between the PA patients and the control group (*p* = 0.077 and *p* = 0.93, respectively). The RVMi was higher in the PA group, at 18.9 (4.9) g/m^2^, versus 13.6 (3.2) g/m^2^ (SD) in the control group (*p* < 0.0001). The RVEDVi was positively correlated with the duration of hypertension (rho = 0.4, *p* = 0.03), and the latter was correlated inversely with the RVEF (rho = −0.47, *p* = 0.009). The RV global longitudinal strain was impaired in PA patients in comparison with the controls (−16.8 (2.5%) versus −19.6 (2.7%), *p* = 0.0001). **Conclusions:** The PA patients exhibited larger RVMi values than the controls. The higher the aldosterone levels were, the higher the observed RVEDVi. Additionally, the longer the duration of hypertension, the higher the observed RVEDVi and the lower the noted RVEF. The PA patients exhibited subclinical RV systolic dysfunction, expressed as impaired RV global longitudinal strain.

## 1. Introduction

Aldosterone excess observed in patients with primary aldosteronism (PA) has a detrimental effect on the cardiovascular system [1,2,3]. There are various mechanisms leading to cardiac dysfunction in PA patients, including increased afterload due to an aldosterone-induced rise in blood pressure (BP). It has been shown that aldosterone causes left ventricular (LV) hypertrophy to a degree which exceeds the impact of hypertension alone. LV hypertrophy is accompanied by cardiac fibrosis, since both hypertension and aldosterone excess are potent factors leading to the development of cardiac fibrosis [4]. This further exacerbates the impaired systolic and diastolic function of the left ventricle. Additionally, aldosterone is a potent proinflammatory agent leading to a vicious cycle of fibrosis and inflammation [3,5,6,7,8,9]. While there is a wealth of data on the impact of aldosterone on the left ventricle, no data exist concerning right ventricular (RV) function in patients with PA [1,3,10,11,12].

The vast majority of research exploring LV structure, function, and remodeling in PA patients used transthoracic echocardiography with all of its benefits [12,13]. Transthoracic echocardiography is the first-line imaging method for heart assessment in patients with hypertension, including patients with PA. It enables accurate evaluation of LV size and function, including but not limited to measurements of cardiac chamber sizes, evaluation of heart valves, and estimation of the LV ejection fraction. Additionally, echocardiography is a reliable tool for the assessment of subclinical systolic dysfunction, using the speckle tracking technique and LV strain calculation. However, echocardiography also poses inevitable limitations [14]. One of them is its relatively impaired ability to assess RV size and function. This is mainly due to the irregular crescent shape of the right ventricle. Three-dimensional echocardiography has an incremental value in this situation; however, RV assessment remains challenging. Cardiac magnetic resonance (CMR) is an ideal tool for the evaluation of the right ventricle free from geometric assumptions [15,16]. It enables reliable and reproducible calculations of RV volumes, ejection fraction, and mass. Additionally, strain analysis is an established method for the detection of subclinical myocardial dysfunction.

Accordingly, we aimed to assess RV structure and function in patients with primary aldosteronism using CMR.

## 2. Materials and Methods

Consecutive patients with PA who underwent a CMR study were included. Exclusion criteria encompassed any contraindications for CMR study (e.g., metallic objects non-compatible with magnetic resonance environment), the administration of a gadolinium-based contrast agent (allergic reaction in the patient’s history or advanced kidney disease with an estimated glomerular filtration rate below 30 mL/min/1.73 m^2^), previous myocardial infarction, suspected or confirmed myocarditis in the patient’s history, severe heart failure, present or suspected chronic coronary syndrome with flow-limiting coronary artery stenosis, malignancy (present or in the patient’s history), and pregnancy. Additionally, we excluded all patients with chronic pulmonary diseases (e.g., chronic obstructive pulmonary disease), pulmonary embolism in their history, more than mild tricuspid regurgitation, or other conditions that may impact RV structure and function.

PA was diagnosed based on the Endocrine Society Clinical Practice Guidelines [17]. In brief, the plasma aldosterone/renin ratio was measured to detect possible cases of PA in the group of patients with suspected PA, namely sustained BP exceeding 150/100 mm Hg at each of 3 measurements performed 1–2 min apart (see below) and obtained on different days; subjects with hypertension resistant to 3 conventional antihypertensive medications, including a diuretic; controlled BP for at least 4 antihypertensive drugs; patients with hypertension and spontaneous or induced by diuretics for hypokalemia; adrenal incidentaloma with concomitant hypertension; hypertension and sleep apnea; hypertension with a family history of cerebrovascular accident or early onset hypertension (below 40 years); and hypertensive first-degree relatives of patients with PA [17]. Subsequently, patients with a positive plasma aldosterone/renin ratio underwent a confirmatory test (a saline infusion test) to definitively confirm or exclude the diagnosis. A saline infusion test was carried out in the seated position. An intravenous infusion of 2 L of 0.9% saline over 4 h was performed [17]. The test was started at 8:00–9:30 AM. Postinfusion plasma aldosterone levels higher than 10 ng/dL confirmed a diagnosis of PA.

All patients were screened with the use of transthoracic echocardiography for the presence of pulmonary hypertension using an estimation of systolic artery pulmonary pressure from continuous wave Doppler measurements [18,19]. The echocardiographic probability of pulmonary hypertension was assessed based on peak tricuspid regurgitation velocity (m/s). A velocity ≤ 2.8 m/s or one not measurable and with no other echo pulmonary hypertension signs indicated a low probability of pulmonary hypertension, while a velocity higher than 3.4 m/s indicated a high echocardiographic probability of pulmonary hypertension. Similarly, a velocity between 2.9 and 3.4 m/s with the presence of other echocardiographic pulmonary hypertension signs indicated a high probability of pulmonary hypertension. All other situations indicated intermediate probability [18,19].

Office blood pressure measurements were made according to 2018 European Society of Cardiology/European Society of Hypertension guidelines for the management of arterial hypertension, valid at that time [20]. Briefly, 3 measurements were performed by a trained nurse using an automated device (Omron 705IT, Omron Co., Kyoto, Japan) 1–2 min apart, and BP was recorded as the average of the last two BP readings. These measurements were preceded by 5 min rest in a seated position in a quiet environment for 5 min. In cases when the first two readings differed by >10 mmHg, additional measurements were recorded. The wideness of a cuff was adjusted to the patient’s arm circumference. The lower edge of the cuff was placed 2 cm above the antecubital fossa.

All CMR studies were performed with a 1.5 T scanner (Avanto fit, Siemens, Erlangen, Germany) with dedicated phased array cardiac coils. At first, several scout images were acquired to plan cardiac axes. Breath hold, ECG-gated balanced steady-state free precession cine images were obtained in the left ventricle long axis, resulting in 2-chamber, 3-chamber, and 4-chamber views. A stack of LV short axis cine images was acquired from the base to the apex covering the entire left and right ventricle. LV and RV end-diastolic volumes, end-systolic volumes, stroke volumes, ejection fractions, and masses were calculated using dedicated software (Syngo.via version: VB80D, Siemens, Erlangen, Germany) after semiautomated delineation of epi- and endocardium in end diastole and end systole. RV end-diastolic, end-systolic, and stroke volumes (RVEDV, RVESV, and RVSV, respectively), as well as RV mass (RVM), were indexed to body surface area—RVEDVi, RVESVi, RVSVi, and RVMi. RV global longitudinal strain (RVGLS) was measured with Segment CMR software version 4.2.1.0 (Medviso, Lund, Sweden) [21]. First, manual delineation of RV endocardial contours in end diastole in 4-chamber view was performed. The tricuspid valve plane was marked. Thereafter, propagation of the contours was performed in all cardiac phases throughout the heart cycle, with manual correction if needed [21]. Other researchers found a high level of agreement in the evaluation of RV global longitudinal strain (GLS) with a high interobserver and interobserver correlation coefficients. Especially, it is highly reproducible (although less than LV strain) when CMR is used to assess RV strain [22]. All CMR studies were analyzed by an experienced cardiologist (M.Ś) with over 15 years of experience in performing and analyzing CMR studies.

A group of sex- and age-matched healthy volunteers served as a control group. This study was approved by the local Ethics Committee (IK.NPIA.0021.61.1887/2020—for study with PA patients and IK.NPIA.0021.68.1995/22 for healthy volunteers), and all participants gave their written informed consent.

All statistical analyses were performed using MedCalc statistical software (version 23.1.7, Ostend, Belgium). The normal distribution of continuous data was tested with the use of the Kolmogorov–Smirnov test. Normally, distributed variables are compared using the Student’s *t*-test for independent samples. If the data had non-Gaussian distribution, they were compared with the use of the Wilcoxon test. Continuous data are presented as means (SD) or medians with interquartile ranges (IQRs), as appropriate. Categorical variables were compared using either Fisher’s exact test or the chi^2^ test, while correlations were tested with the use of either the Pearson correlation coefficient (r) or Spearman’s rank correlation coefficient (rho). All tests were two-sided. A *p*-value equal to or smaller than 0.05 denoted statistical significance.

## 3. Results

Out of 32 patients with PA who underwent CMR, 30 patients were included in the final analyses. The reasons for excluding two patients were as follows: in one patient image quality was poor and related to claustrophobia; the second patient was excluded in order to create a cohort homogenous to our previous study [23].

The groups did not differ in terms of age and sex distribution (Table 1). In the PA group, mean systolic BP was 160.1 (11.1) mmHg and mean diastolic BP was 97.8 (13.4) mmHg. All individuals in the control group were normotensive. In all PA patients there was low echocardiographic probability of pulmonary hypertension according to the then-valid 2015 European Society of Cardiology/European Respiratory Society Guidelines for the diagnosis and treatment of pulmonary hypertension [18].

Neither RVEDVi or RVEF differed between PA patients and the control group (Table 2). A significant difference was observed when we compared RVMi, which was higher in the PA group (Table 2). RVMi did not exceed the upper reference limit in all controls [24]. In the PA group, however, two women exhibited RVMi that was slightly above the upper reference limit. RV mass-to-volume ratio was greater in patients with PA than in control subjects (Table 2).

We further studied associations of aldosterone levels with CMR-derived RV parameters. Neither the RVMi or the RVEF correlated with aldosterone levels (rho = 0.15, *p* = 0.36 and rho= −0.17, *p* = 0.37). On the contrary, we found a weak positive correlation between RVEDVi and aldosterone concentration (rho = 0.5, *p* = 0.005).

Strain analysis of the right ventricle revealed better RVGLS in controls when compared to PA patients (Table 2).

**Figure 1:**
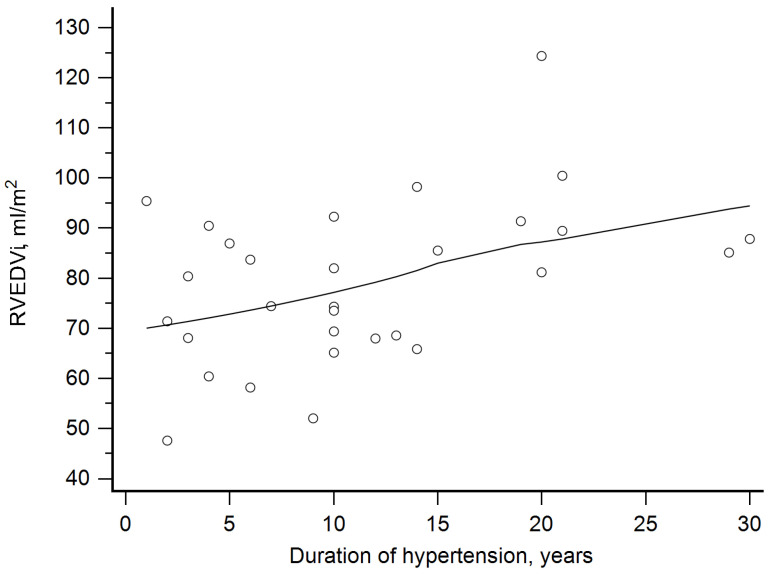
Positive correlation between right ventricular end-diastolic volume index (RVEDVi) and duration of hypertension (rho = 0.4, *p* = 0.03).

**Figure 2:**
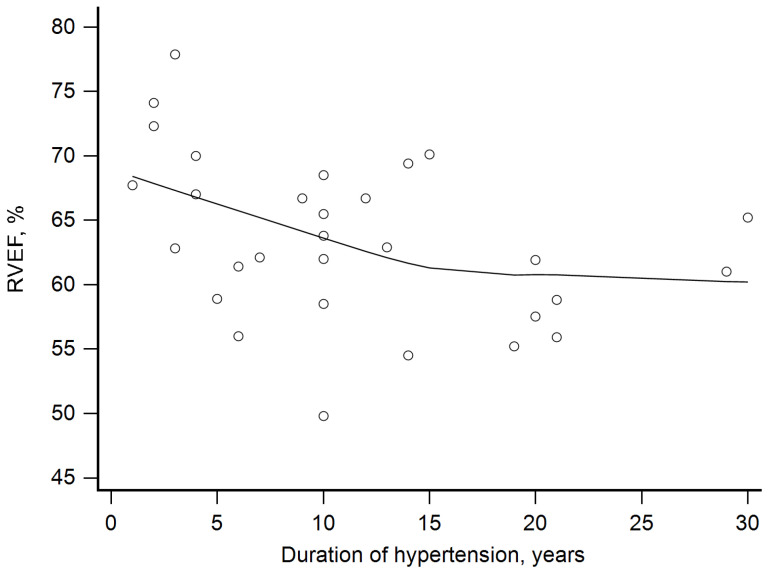
Negative correlation between right ventricular ejection fraction (RVEF) and duration of hypertension (rho= −0.47, *p* = 0.009).

Finally, we assessed relationships between the hypertension duration and RV parameters. RVMi did not correlate with the duration of hypertension. RVEDVi was positively correlated with the duration of hypertension (rho = 0.4, *p* = 0.03, Figure 1). The latter was correlated inversely with RVEF (rho= −0.47, *p* = 0.009, Figure 2). We have also calculated partial correlation coefficients with age and plasma aldosterone level as covariates. The correlation of both RVEF and RVEDVi with the duration of hypertension remained significant: for RVEF, the coefficient was −0.52 and *p* = 0.004; for RVEDVi, the coefficient was 0.39 and *p* = 0.04.

The left ventricular parameters derived from CMR imaging in PA patients and the control group are presented in Supplementary Table S1.

## 4. Discussion

To the best of our knowledge, this is the first study assessing right ventricular structure and function with the use of CMR in PA patients. The main findings of our study are as follows:

(i) The longer duration of hypertension, the higher the observed RVEDVi, and the longer duration of hypertension, the lower RVEF was noted.
(ii) Although RVMi was within the normal range in all but two patients, it was greater in PA patients than in controls.
(iii) Patients with PA exhibited unfavorable remodeling confirmed by higher RV mass-to-volume ratio when compared to healthy volunteers.
(iv) PA patients exhibit subclinical RV systolic dysfunction expressed as impaired RV global longitudinal strain.

Previous studies demonstrated that in patients with arterial hypertension, increased pulmonary pressure may be present [25]. This in turn may lead to RV dilatation and dysfunction [26]. Nevertheless, all patients in our study had low echocardiographic probability of pulmonary hypertension, as assessed by transthoracic echocardiography. A longer hypertension duration was associated with an increased RVEDVi and simultaneously with a lower RVEF in our study. In other words, patients with long-term hypertension burden demonstrated a dilated right ventricle (although still within the normal range in the vast majority of patients) and a lower RVEF (again, still within the normal range in all patients). This observation is intriguing, since other authors suggest that the development of RV heart failure is caused by severe associated pulmonary hypertension, which gradually increases with increasing severity of hypertension (from grade I to grade III) [27]. The same group of investigators demonstrated that both resting and exercise for pulmonary vascular resistance are increased in patients with uncomplicated hypertension [28]. As already mentioned, all patients in our study were screened for the presence of pulmonary hypertension, and none had an intermediate or high echocardiographic probability of pulmonary hypertension. Despite this fact, the patients in our study exhibited lower RVEF with increasing duration of hypertension. Apart from the indirect effects of aldosterone on the right ventricle (the development of increased pulmonary artery pressure, increased afterload, and progression of RV heart failure often occurring as a consequence of left-sided heart failure), the direct detrimental effect of aldosterone excess on the right ventricle should be taken into consideration [29,30]. Mineralocorticoid receptors have been detected in the human myocardium [31]. However, their clinical relevance remains controversial. Nevertheless, there are several mechanisms by which aldosterone may induce right ventricular dysfunction, namely fibrosis, RV remodeling, inflammation, and increased oxidative stress. RV scarring (fibrotic tissue formation) is a potent mechanism leading to both systolic and diastolic dysfunction. As mentioned below, in the limitations of this study, in a normal clinical scenario CMR is not able to reliably assess the presence and extent of RV fibrosis in non-severely hypertrophied RV. Partial volume effect averaging the signal of RV wall, blood pool, and surrounding tissues makes the evaluation of the right ventricle challenging. However, emerging CMR techniques like using high-resolution imaging may overcome these limitations related to thin-walled RV walls [32]. In particular, high-resolution T1 mapping developed specifically to evaluate the right ventricle seems to be very promising [32]. Using pre-contrast and post-contrast high-resolution T1 mapping, one is able to assess the diffuse RV fibrosis expressed as an expansion of the extracellular volume. Additionally, acquiring T1 mapping images in end systole may also be beneficial in assessing thin RV wall. High-resolution T1 mapping techniques should be explored in future studies.

Partial volume effect may limit the reliability of RV mass calculations. Additionally, RVM is usually not quantified in normal routine clinical scenarios [16]. However, virtually all papers on the normal reference values for right and left ventricular quantification by CMR provide information on the reference ranges for RVM [24]. This proves that reliable RVM calculation using CMR is feasible even in thin RV walls in healthy volunteers. Additionally, the method used for cine image acquisition (namely, a slice thickness of 8 mm) limits partial volume effects [33]. A previous study showed that the reproducibility of RV measurements is adequate to detect RV hypertrophy [34]. Espe et al. demonstrated a simple post-processing algorithm for the correction of partial-volume effects [35]. This warrants further studies aimed to assess whether this method may further improve the reproducibility of RVM calculation in hypertensive patients.

We demonstrated that patients with PA had greater RVMi than healthy volunteers. Notably, in the vast majority of them (93%), RVMi was still within the normal reference range. Interestingly, while RVMi and LVMi showed positive correlation (rho = 0.44, *p* = 0.01) in controls, in PA patients we observed a lack of correlation between RVMi and LVMi (rho = 0.31, *p* = 0.09) proving disproportional hypertrophy of the right and left ventricles. As mentioned above, it seems unlikely that increased pulmonary artery pressure contributed to an increase in the RVMi. Ventricular interaction is another cause of RV “hypertrophy” and, in general, RV remodeling in patients with PA [25]. The interventricular septum serves as a medium transferring hemodynamic changes from the left to the right ventricle. A higher RV muscle mass in PA patients can also be attributable to the direct impact of aldosterone on RV cardiomyocytes [36].

The RV mass-to-volume ratio, also known as the remodeling index, reflects the structural adaptation of the right ventricle by thickening RV walls to compensate for increased workload [37]. While in the physiological response to increased workload, the RV mass-to-volume ratio remains stable (reflecting harmonious increase in ventricular mass and volume), in the pathological response to pressure or volume overload, it increases as a result of disproportional ventricular hypertrophy in relation to its volume [37]. While the exact normal range can vary slightly depending on the study and imaging technique, a general guideline suggests that a normal RV mass-to-volume ratio is typically around 0.18–0.19 g/mL, a value which is similar to the one obtained in healthy volunteers in our study [38]. On the other hand, RV mass-to-volume ratio exceeding 0.2 g/mL proved to be associated with a subsequent increase in the RVEDVi [39]. In patients with PA, this ratio was 0.25 g/mL on average, indicating that a further increase in the RV volume can be expected. However, this could only be confirmed in a longitudinal study assessing RV size constantly over time.

Finally, we demonstrated that patients with PA and hypertension show a maladaptive change, not only in the structure, but also in the function of the right ventricle. Although RVEF was similar to healthy volunteers, RVGLS showed a slight impairment of RV systolic function. This subclinical decrease in RV systolic function may be attributable to RV fibrosis observed in animal models of aldosterone/salt-induced hypertension [29,30]. Several previous studies demonstrated impaired RV strain in patients with arterial hypertension. These studies are summarized in a comprehensive review paper by Tadic and colleagues [40]. However, none of these studies employed CMR as a primary tool in assessing RVGLS. Additionally, none of them included patients with PA. Of note, it has been demonstrated that RVGLS was gradually impaired from individuals with optimal BP (24 h SBP < 120 mmHg), across subjects with a high–normal BP (120 mmHg ≤ 24 h SBP ≤ 130 mmHg) to patients with hypertension (24 h SBP > 130 mmHg) [41]. This data indicates that there is a spectrum of impaired RV strain depending on the severity of BP raise. One may speculate that in patients with PA, who represent often high BP values (in our study, systolic office BP in the PA group was 160.1 mmHg on average), this deterioration of RV systolic function may be even more severe. Additionally, RV free wall strain and 3D-echocardiography-derived RV stroke volume proved to be independent predictors of peak oxygen consumption in cardio-pulmonary exercise tests [42]. Both parameters, namely RV strain and RV stroke volume, may also be derived from CMR study, with higher accuracy and reproducibility. However, investigations using CMR and cardiopulmonary exercise testing in PA patients are lacking. This issue should be explored in further studies.

Previous studies in various populations (e.g., heart failure, pulmonary embolism, pulmonary hypertension) proved that RVGLS is a valuable prognostic marker [43,44,45]. Lower RVGLS values are associated with worse outcomes. The same association has been demonstrated in hypertensive patients [46,47]. It is therefore plausible that in PA patients RVGLS carry prognostic information. Further longitudinal studies are necessary to assess the prognostic value of RVGLS in this population. Additionally, it should be elucidated whether timely interventions like adrenalectomy or prompt initiation of pharmacological treatment can improve RVGLS in PA patients and affect prognosis.

Several limitations of our study should be acknowledged, including its low sample size. However, there are only a few studies assessing cardiac structure and function using CMR in patients with PA [4,11,48,49,50,51,52]. Moreover, studies evaluating the right ventricle in this population with the use of CMR are lacking. Although there are data on the right ventricle in patients with arterial hypertension, they utilized echocardiography as a primary tool, providing data on RV size and function [25,27,37,40,53,54]. As already mentioned, echocardiography has inevitable limitations in assessing the right ventricle, and CMR is able to overcome these limitations. In contrast to patients with pulmonary arterial hypertension and significant RV hypertrophy, assessment of RV fibrosis in non-hypertrophied RV wall in CMR is very challenging due to the low thickness of normal RV wall and the relatively low spatial resolution of CMR images. Therefore, we did not assess late gadolinium enhancement in the right ventricle.

Finally, it remains unknown whether observed alterations in RV structure and function are more pronounced in patients with PA when compared to essential hypertension patients. This needs to be elucidated in further studies implementing control groups including patients with essential hypertension matched by BP values.

## 5. Conclusions

In conclusion, PA patients exhibited a larger RVMi than controls. The higher the aldosterone levels were, the higher the RVEDVi. Additionally, the longer the duration of hypertension, the larger the right ventricle and the lower the RVEF noted. PA patients exhibited subclinical RV systolic dysfunction, expressed as impaired RV global longitudinal strain.

## Figures and Tables

**Table 1:** Baseline characteristics of patients with primary aldosteronism and the control group.

	Primary Aldosteronism Patients (n = 30)	Controls (n = 30)	*p*-Value
Age, years	51.0 (10.4)	50.7 (10.6)	0.88
Sex, males	50%	50%	1.0
Duration of hypertension, years	10.0 (5.0–15.0)	–	–
Aldosterone, pg/mL	263.5 (175.0–473.0)		
Renin, pg/mL	1.8 (0.78–5.0)		
Aldosterone renin ratio	168 (58–587)		
eGFR, mL/min/1.73 m^2^	100 (83–105)		
Potassium, mmol/L	3.63 (0.51)		
Number of antihypertensive medications, n	4 (3–5)	–	–
Medications at the time of CMR		–	–
RAS blocker, n (%)	28 (93.7)	–	–
ACE inhibitors, n (%)	14 (46.7)	–	–
ARB, n (%)	14 (46.7)	–	–
Calcium channel blocker, n (%)	24 (80)	–	–
Beta-blocker, n (%)	22 (73.3)	–	–
MRA, n (%)	16 (53.3)	–	–
Diuretics, n (%)	12 (40)	–	–
Thiazide/Thiazide-like, n (%)	10 (33.3)	–	–
Loop, n (%)	2 (6.7)	–	–
Alpha-blocker, n (%)	11 (36.7)	–	–

ACE, angiotensin-converting-enzyme; ARB, angiotensin receptor blocker; BSA, body surface area; CMR, cardiac magnetic resonance; eGFR, estimated glomerular filtration rate according to the Chronic Kidney Disease Epidemiology Collaboration 2021 equation; MRA, mineralocorticoid receptor antagonist; RAS, renin–angiotensin system; data are means (SD), medians (interquartile ranges). or numbers (percentages).

**Table 2:** Right ventricular parameters derived from cardiac magnetic resonance imaging in primary aldosteronism patients and the control group.

	Primary Aldosteronism Patients (n = 30)	Controls (n = 30)	*p*-Value
RVEDVi, mL/m^2^	79.0 (16.0)	72.0 (13.3)	0.077
RVEDVi exceeding the upper reference range, n (%)	1 (3.3%)	0	1.0
RVESVi, mL/m^2^	29.4 (9.7)	26.7 (8.7)	0.27
**RVSVi, mL/m^2^**	**49.6 (8.5)**	**45.4 (6.1)**	**0.03**
RVEF, %	63.5 (6.4)	63.7 (6.2)	0.93
RVEF below the lower reference range, n (%)	0	0	1.0
**RVMi, g/m^2^**	**18.9 (4.9)**	**13.6 (3.2)**	**<0.0001**
RVMi exceeding the upper reference range, n (%)	2 (6.7%)	0	0.49
**RV mass-to-volume ratio, g/mL**	**0.25 (0.08)**	**0.19 (0.04)**	**0.001**
**RVGLS, %**	**−16.8 (2.5)**	**−19.6 (2.7)**	**0.0001**

Data are means (SD), RVEDVi, right ventricular end-diastolic volume index; RVESVi, right ventricular end-systolic volume index; RVEF, right ventricular ejection fraction; RVGLS, right ventricular global longitudinal strain, RVMi, right ventricular mass index; and RVSVi, right ventricular stroke volume index.

## Data Availability

The datasets generated and/or analyzed during the current study are available from the corresponding author on reasonable request. The data are not publicly available due to institutional and ethical restrictions.

## Supplementary Materials

The following supporting information can be downloaded at: https://www.mdpi.com/article/10.3390/jcm14155367/s1, Table S1: Left ventricular parameters derived from cardiac magnetic resonance imaging in primary aldosteronism patients and the control group.

## Abbreviations

The following abbreviations are used in this manuscript:

BP	Blood pressure
BSA	Body surface area
CMR	Cardiac magnetic resonance
eGFR	eGFR, estimated glomerular filtration
IQR	Interquartile range
LV	Left ventricular
PA	Primary aldosteronism
RV	Right ventricular
RVEDVi	Right ventricular end-diastolic volume index
RVEF	Right ventricular ejection fraction
RVESVi	Right ventricular end-systolic volume index
RVGLS	Right ventricular global longitudinal strain
RVMi	Right ventricular mass index
RVSVi	Right ventricular stroke volume index
SD	Standard deviation
