# Supplementary material for: Right Ventricular Structure and Function in Patients with Primary Aldosteronism: A Cardiac Magnetic Resonance Study
Source: J Clin Med. 2025 Jul 29;14(15):5367. doi: 10.3390/jcm14155367 (PMC12346917; doi:10.3390/jcm14155367)
Supplement: Supplementary file 1 [file jcm-14-05367-s001.zip › jcm-3731324-supplementary.pdf]

Supplementary Table S1. Left ventricular parameters derived from cardiac magnetic resonance imaging in primary aldosteronism patients and the control group.

	**Primary aldosteronism patients (n=30)**	**Controls (n=30)**	***P*-value**
**LVEDVi, ml/m²**	**82.9 (15.9)**	**73.9 (12.9)**	**0.019**
LVEDVi exceeding the upper reference range, n (%)	2 (6.7%)	0	0.49
**LVESVi, ml/m²**	**33.2 (9.9)**	**27.4 (7.5)**	**0.01**
LVSVi, ml/m²	49.7 (7.4)	46.1 (7.0)	0.09
**LVEF, %**	**60.6 (5.2)**	**63.4 (5.1)**	**0.04**
LVEF below the lower reference range, n (%)	3 (10%)	0	0.24
**LVMi, g/m²**	**83.6 (24.2)**	**58.4 (10.2)**	**<0.0001**
**LVMi exceeding the upper reference range, n (%)**	**26 (86.7%)**	**0**	**<0.0001**
**LV mass-to-volume ratio, g/ml**	**1.0 (0.27)**	**0.82 (0.13)**	**0.0005**
**LVGLS, %**	**-18.5 (2.5)**	**-21.8 (2.7)**	**<0.001**

Data are means (SD)

LVEDVi, left ventricular end-diastolic volume index; LVESVi, left ventricular end-systolic volume index; LVEF, left ventricular ejection fraction; LVGLS, left ventricular global longitudinal strain, LVMi, left ventricular mass index; LVSVi, left ventricular stroke volume index
